# A Runway Safety System Based on Vertically Oriented Stereovision

**DOI:** 10.3390/s21041464

**Published:** 2021-02-20

**Authors:** Dawid Gradolewski, Damian Dziak, Damian Kaniecki, Adam Jaworski, Michal Skakuj, Wlodek J. Kulesza

**Affiliations:** 1 Bioseco Sp. z. o. o., Budowlanych 68, 80-298 Gdansk, Poland; damian.dziak@bioseco.com (D.D.); damian.kaniecki@bioseco.com (D.K.); adam.jaworski@bioseco.com (A.J.); 2 Department of Mathematics and Natural Sciences, Blekinge Institute of Technology, 371 79 Karlskrona, Sweden; wlodek.kulesza@bth.se; 3 Ekoaviation, ul. Piecewska 30B/16, 80-288 Gdansk, Poland; ekoaviation@michalskakuj.com

**Keywords:** bird monitoring, bird strike, distributed computing, runway safety, localization, Internet of Things, environmental sustainability, monitoring of avifauna, safety system, stereovision, visual sensor network

## Abstract

In 2020, over 10,000 bird strikes were reported in the USA, with average repair costs exceeding $200 million annually, rising to $1.2 billion worldwide. These collisions of avifauna with airplanes pose a significant threat to human safety and wildlife. This article presents a system dedicated to monitoring the space over an airport and is used to localize and identify moving objects. The solution is a stereovision based real-time bird protection system, which uses IoT and distributed computing concepts together with advanced HMI to provide the setup’s flexibility and usability. To create a high degree of customization, a modified stereovision system with freely oriented optical axes is proposed. To provide a market tailored solution affordable for small and medium size airports, a user-driven design methodology is used. The mathematical model is implemented and optimized in MATLAB. The implemented system prototype is verified in a real environment. The quantitative validation of the system performance is carried out using fixed-wing drones with GPS recorders. The results obtained prove the system’s high efficiency for detection and size classification in real-time, as well as a high degree of localization certainty.

## 1. Introduction

The first collision of a bird with an aircraft, so-called bird strike, was reported in 1905, and then, in 1912 the first fatality was noted [1]. Since then, the number of cases has risen, presenting a significant threat to flight safety and causing a number of tragic accidents worldwide [2]. In 2020, purely in the USA, over 10,000 bird strikes were reported [3]. The reports show that the average bird strike rate, which is counted per 10,000 flights, increased from 11 in the year 2011 to 33 in the year 2017 [4]. According to the International Civil Aviation Organization (ICAO), most of the bird strikes occur during the approach, 33%, take off, 31%, and landing, 26%, which means that 90% of incidents occur in the airspace under the airport’s legal responsibility [4]. The administration and legal regulations introduced by the ICAO and European Union Aviation Safety Agency (EASA) oblige each airport to minimize the bird and wildlife strike risk, under Wildlife and Hazard Management (WHM) [5,6].

Currently, different techniques and methods allowing the mitigation of bird strike risk such as the ornithological observations and radar based solutions [7] are the most widespread at medium and large airports. There are also some attempts to develop vision based monitoring systems [8,9,10]. However, enhancing automation and to improve the system performance levels of WHM in terms of detection efficiency and localization accuracy is a research challenge.

To meet the requirements of a market tailored product, which may be customized to any small and medium size airport, a stereovision based real-time solution embedded into the Internet of Things (IoT) and a distributed computing paradigm is proposed. A new stereovision method with the cameras’ optical axes freely oriented is modeled, evaluated, and implemented in a prototype. The real-time field verification at an airport runway using drones with a GPS recorders shows the system’s capacity to detect moving objects at a range of 300 m and to localize them within the required accuracy of 10%. It is proven that the proposed solution is able to classify detected objects into one of three size categories corresponding to bird size.

## 2. Background and Related Works

The problem of bird strikes is multifaceted and can be approached from the point of view of sustainable development, economy, law, and technology.

### 2.1. Non-Technological Approaches

The non-technological aspect of the presented solution could be analyzed from the perspective of bird presence near the airports, as well as the legal and financial consequences of potential bird strikes.

Increasing volumes of air traffic and the adaptation of some bird species to the living conditions in the vicinity of urban areas, which also increases their activity around airports, are the main causes of the increase in bird collisions [11]. Birds have modified their behavior and learned to tolerate the presence of both humans and man-made structures including air traffic and accompanying noises. Therefore, it is getting more difficult to control or limit their presence in the airport’s vicinity [12].

The problem with the increasing bird strike rate was noted by national and international organizations including the ICAO [13], the World Birdstrike Association (WBA), and states’ aviation authorities [14,15]. These organizations are responsible for sharing information and experiences, as well as for the development of the best practices regarding collision prevention. Currently, environmental monitoring of airports is regulated by the EASA [5] and the ICAO [6]. There are also national civil and military authorities and organizations responsible for aviation safety, like the Civil Aviation Authority [16] in Poland or the Swedish Civil Aviation Administration (Luftfartsverket) [17] in Sweden, who are responsible for wildlife risk management.

The data analysis performed by the ICAO and the EASA shows the critical areas where most of the accidents occur [18]:

- Ninety percent of collisions are below an altitude of 150 m;
- Sixty-one percent of events are at heights of less than 30 m;
- Eight percent of collisions are at an altitude above 900 m and are outside the aerodrome area;
- Seventy-five percent of accidents happen during the day.

The bird strikes with the windshield and the engine of the aircraft are the most dangerous and the most frequent events [19]. These damages cost over $200 million annually [20] purely in the USA and up to $1.2 billion worldwide [21].

### 2.2. Technological Approaches

So far, the most widespread solutions for bird strike prevention at large and medium size airports are still the eye observation of the runway. At many airports, various methods such us trained dogs, falconry, pyrotechnics, and green lasers are used as the most effective tools. Sometimes, deterrents are also installed, which emit predator or banging cannon sounds [20].

There have been a number of attempts to develop reliable autonomous bird detection and localization systems [10,22]. Besides the aforementioned automation of WHM at airports [23], the bird preservation at wind farms [10,24] and autonomous analysis of migrant bird behavior [25,26] are the main application fields.

Mainly, there are two types of sensors used for bird detection: radar [27,28] and vision cameras [9]. One of the first bird detection systems, which used the radar technology, was developed [29,30] in the early 1950s. Since then, the radar based solutions have improved their capabilities of bird detection in wide observation areas. Because of their capacity to estimate the bird’s position, velocity, and movement [31], they have become widely used in airports [32]. Radar systems for bird detection are characterized by long-range detection [33] in any weather and light conditions [34,35]. It is worth noting that the radar based solutions require additional permissions for emission in the frequency band, which should not disrupt the airport’s flight control systems [31].

The vision based solutions can be split into two groups: mono- and stereo-scopic systems. Whereas monoscope systems are able to detect the birds [26,36] and identify particular species [37], the stereoscopic systems additionally allow bird localization and size estimation [10,37,38].

The growth of CPU and GPU capabilities allows the application of advanced algorithms, which are more reliable in moving object detection [39] and identification [40,41]. The parallel enhancement of the resolution of image sensors and the advance in optics make it possible to detect and identify even small objects from far distances [10,26,39,42].

The core component of each vision based system is a detection algorithm. The bird detection in the video stream can be made using motion detection [22,25,43], AI based identification [26,44,45,46,47,48,49], or a combination of both [10,38]. Whereas the motion detection algorithms allow the reduction of the computational complexity of the safety system [50], the application of AI methods allows bird identification [48,49] and the reduction of false positive rates [10]. From the AI based solutions, the Convolutional Neural Networks (CNNs) [51,52,53] outperform other methods, for instance the Haar feature based cascade classifier [45,54] or Long Short-Term Memory (LSTM) [48]. The most recent studies reported that dense CNN [54] shows good feature extraction capabilities allowing for bird identification [49,55], and after 100 epochs, the system reaches near 99% accuracy. Other CNNs, implemented in distributed computing and IoT paradigms, allow the system to ensure 99.8% precision with 99.0% recall in bird identification with real-time performance [10]. This allows the development of a reliable vision based safety system at airports.

There are several examples of vision based systems allowing WHM at airports. Chen et al. proposed an automatic bird-blocking network and intelligent bird-repelling system [56]. The proposed algorithm with the use of IoT technology allowed automatic repelling, which minimizes the habituation effect [56]. The company Pharovision developed a commercially available system that is based on the infrared camera and allows scanning of the ground and the air, day and night [9]. Using the FLIR and CCTV cameras, their system detects and tracks even a single bird from up to 7 km [9]. Another complex system allowing multiple bird detections and repelling is provided by Volacom [8]. Detection is supported by thermal and stereovision cameras, which in real-time scan the airport’s vicinity for flying objects [8]. An additional acoustic module focuses a deflection sound signal at the targeted birds, to deter them up to 300 feet [8].

After detection, an automatic repellent method could be applied to minimize the bird strike risk. One of the first repelling methods tested in various scenarios was pulsing light [23]. This method was successfully used at an airport [57], other man-made structures [58], and wind farms [10]. Since the year 2015, pulsing light at 2 Hz in the landing light system has been recommended by the Federal Aviation Administration (FAA) and successfully used in airplanes and helicopters as a tool, allowing a substantial drop in bird collisions [59]. The other solution mounted in airports near the runway is large screens displaying a specific visual sight [60].

To deter a bird, a loud sound can also be used. Bishop et al. [61] showed that high frequency sound in the ultrasonic range above 20 kHz is ineffective and therefore has no biological basis for its use. In [62], the authors combined the effect of sound between 90 dB and 135 dB and a frequency of 2 kHz with white light. To reduce the habituation effect of the repellent method, the particular deterrent method used should vary and be implemented as rarely as possible [61].

## 3. Problem Statement, Objectives, and Main Contributions

As the survey of related works shows, there is a need for a reliable and cost-effective system mitigating the collision of avifauna with airplanes around airport runways. The biggest drawback of existing solutions, mostly based on stereovision, is their basically horizontally oriented Field of View (FoV), limiting the observation area and therefore requiring multiplied installations, which are heavy and costly.

The main objective of the paper is to determine the hardware and software structures of a stereovision based detection system for monitoring space over the airport runway to identify, localize, and classify moving objects. Such a highly reliable real-time system has to assure a wide range observation area without compromising its size and price, whilst also providing a wide range of customizability.

The proposed hardware configuration is composed of two cameras coupled in stereovision mode, wherein the first and second cameras are oriented with their optical axes of an angle α to the base line, wherein α is a substantially non-right angle. The cameras could be equally rotated to any direction to cover the selected observation area, which can be horizontal, vertical, or even oblique. The system software configuration is based on IoT technology, the distributed computing concept, and deep learning algorithms to ensure real-time operation mode.

The user-driven design methodology is used to provide a market tailored solution that may be customized to any small and medium size airports. The proposed solution was modeled and optimized using MATLAB software. The system prototype was installed in an real environment and verified using fixed-wing drones with GPS recorders.

## 4. System Design

The proposed avifauna monitoring system for runways was designed based on the User-Driven Design (UDD) methodology presented in [63,64]. Besides airport stakeholders and designers, the design process involved several authorities such as ornithologists and experts in aviation laws. Furthermore, future users, who contributed to the design, were falconers, airport security and safety staff, pilots, maintenance service workers, and environmental workers.

It is beyond a doubt that such a system is demanded to minimize the collision risk due to:

- passengers and staff safety;
- wildlife protection;
- financial consequences related to damages and delays;
- the legal, administrative, and marketing consequences of a potential catastrophe.

To achieve the listed goals, the designed system needs to fulfill the following functionalities and constraints:

- to detect and localize suspected moving objects within the customizable safe zones and to do this with high reliability and low positioning uncertainty;
- to distinguish individual, multiple, or flocks of birds simultaneously;
- to work in real time with a very short detection latency;
- to ensure that bird risk management has no side effects;
- to eliminate the human factor by autonomous monitoring and repelling methods;
- to ensure the affordability of the system including that the system price, cost of installation, and cost of maintenance are acceptable for small airports;
- to facilitate and automate the reporting process recommended by the ICAO and the EASA regulations.

General and itemized functionalities and particular related constraints along with selected technologies and algorithms are summarized in Table 1. The motivation analysis of the technologies and algorithm selection is beyond the scope of this paper. However, it can be observed that the system is based on stereovision and the distributed computing and IoT concepts. The chosen algorithms belong to the machine learning and AI categories. The details of the applied solutions are presented in Section 5 and Section 6.

## 5. Modeling

**Figure 1:**
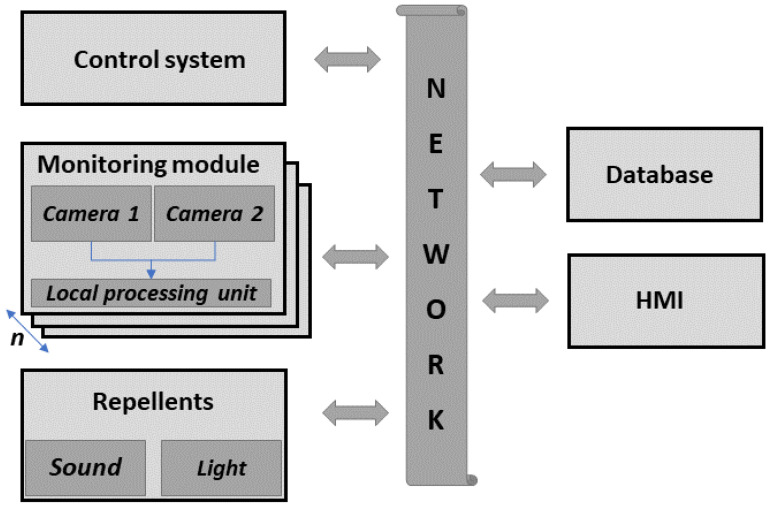
Block diagram illustrating the system conceptualization.

The system conceptualization is presented in Figure 1. Since there is a need to cover a wide observation space, the system consists of several *monitoring modules* and other subsystems inter-connected via the *network*, which becomes a central component of the proposed structure. On the network’s right side, there are components allowing the user to interact with the system. On its left side, there are the *control unit* along with the sensors and actuators responsible for data acquisition and system reactions. The system is based on the IoT and distributed computing concepts [50], facilitating communication between modules and providing easy access to the storage data through the intuitive *GUI*.

The system can be deployed along the runway and consists of the *control system*, *monitoring modules*, and *repellent part*. Each monitoring module includes the stereovision sensing unit and Local Processing Unit (LPU) responsible for motion detection, object identification, and localization. Data from all *monitoring modules* are sent to the *control system*, where the detected object is cropped from the picture and processed, and a decision is made about using the *repellent part*. The *control system* handles the connection with several *monitoring modules*, *repellent parts*, the *database*, and the Human Machine Interface (HMI).

In the *database*, the data of the detected events such us the bird’s position, estimated flight-path, images, and movies, as well as info regarding any actions undertaken are collected. Archived data are accessible through the HMI such as web and mobile applications. The HMI can be also used to manually activate the *repellents part* and maintain/test the system.

### 5.1. Model of the Modified Stereovision Method

**Figure 2:**
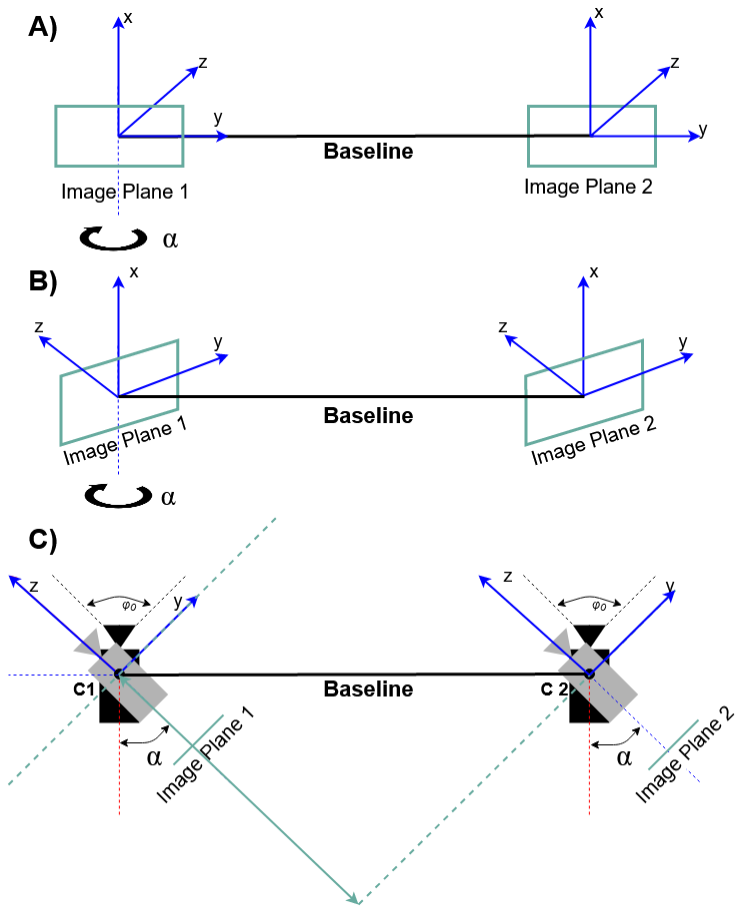
Stereovision model: (**a**) classical; (**b**) with rotated image planes; (**c**) illustration of the cameras’ alignment for the modified stereovision.

A stereovision based *monitoring module* oversees a selected runway zone. The proposed new solution ensures that the observation space is freely selectable through the adjustment of the cameras’ optical axes by changing the orientation of their angles α(*z*); see Figure 2.

In classical stereovision, the cameras’ optical axes are perpendicular to the baseline, *B*, where the baseline is the line segment connecting the cameras’ centers. Then, the baseline and the cameras’ image planes are placed on the same Euclidean plane; see Figure 2a. In the proposed modified stereovision method, the cameras’ optical axes are set at an angle α with respect to the baseline, in such a way that the cameras’ image planes are placed on two parallel planes, as shown in Figure 2b. The cameras’ alignment is presented in Figure 2c [65,66].

**Figure 3:**
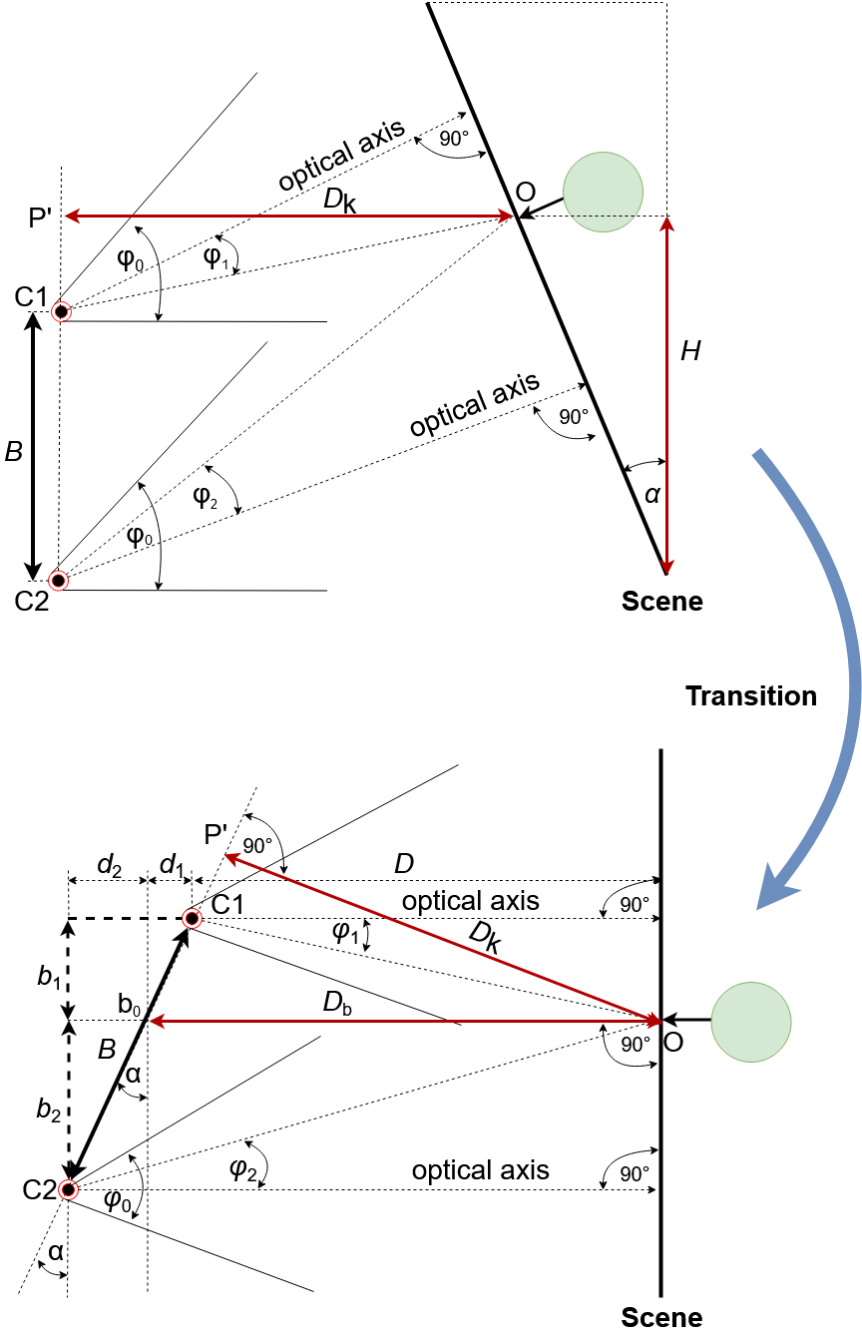
Definition of the variables and basic system settings.

To understand the extraction of the 3D features, the coordinates of the modified stereovision system can be transformed using some geometric features. The transformation is carried out in relation to the first camera C1 (see Figure 3) in such a way that the coordinates and the scene are shifted by the rotation angle α. Using the geometrical transformation, the modified mathematical model of the method can be delivered using the variables and parameters defined in Table 2.

#### 5.1.1. Distance Measurement Using Modified Stereovision

The distance, *D*, from the first camera C1 to the plane of the object, wherein the plane of the object is a plane perpendicular to the optical axes of the cameras, is equal to the distance $D_{k}$ from the object to the baseline, $D=D_{k}$. From the basic geometry, one may also find that $D=D_{b}-d_{1}$ and $B\times \cos\alpha =b_{1}+b_{2}$, where $b_{1}$ and $b_{2}$ are defined as:

(1) $$\left\{\begin{array}{c} b_{1}=D\tan\varphi_{1}\\ b_{2}=D\tan\varphi_{2} \end{array}\right.$$

Then, after substitution:

(2) $$B\times \cos\alpha =\left(D_{b}-d_{1}\right)\times \tan\varphi_{1}+\left(D_{b}+d_{2}\right)\times \tan\varphi_{2}$$

Knowing that $d_{2}=B\times \sin\alpha -d_{1}$:

(3) $$\begin{array}{cc} B\times \cos\alpha = & \left(D_{b}-d_{1}\right)\times \tan\varphi_{1}+\\ & \left(D_{b}+B\times \sin\alpha -d_{1}\right)\times \tan\varphi_{2} \end{array}$$

which could be simplified to:

(4) $$B\times \cos\alpha =\left(D_{b}-d_{1}\right)\times \tan\varphi_{1}+\left(D+B\times \sin\alpha \right)\times \tan\varphi_{2}$$

From this, distance, *D* can be calculated as:

(5) $$D=\frac{B\times (\cos\alpha -\sin\alpha \tan\varphi_{2})}{\tan\varphi_{1}+\tan\varphi_{2}}$$

The angles $\varphi_{1}$ and $\varphi_{2}$ may be found from the relationships:

(6) $$\frac{y_{1}}{\frac{y_{0}}{2}}=\frac{\tan\varphi_{1}}{\tan\frac{\varphi_{0}}{2}}$$

(7) $$\frac{-y_{2}}{\frac{y_{0}}{2}}=\frac{\tan\varphi_{2}}{\tan\frac{\varphi_{0}}{2}}$$

Then, distance *D* can be defined as:

(8) $$D=\frac{B\times \cos\alpha \times y_{0}}{2\times \tan\frac{\varphi_{0}}{2}\times \left(y_{1}-y_{2}\right)}+\frac{B\times \sin\alpha \times y_{2}}{\left(y_{1}-y_{2}\right)}$$

which, for α=0, gives the distance for classical stereovision:

(9) $$D=\frac{B\times y_{0}}{2\times \tan\frac{\varphi_{0}}{2}\times \left(y_{1}-y_{2}\right)}$$

Knowing the distance *D* and the angle $\varphi_{0}$, the object altitude could be calculated using the formula:

(10) $$H=D\times \cot\left(\frac{\varphi_{0}}{2}+\varphi_{2}\right)$$

where $\varphi_{2}$ can be found from (7).

#### 5.1.2. Quantization Uncertainty of the Distance Measurement

The distance *D* defined by (8) is a non-linear discrete function of $y_{0}$, *B*, $\left(y_{1}-y_{2}\right)$, $y_{2}$, and $\varphi_{0}$. The measurement uncertainty, ΔD, determined by the exact differential method [10,67,68], can be expressed by:

(11) $$\begin{array}{cc} \Delta D= & \pm \left(\frac{y_{0}\times \cos\alpha }{2\times \tan\frac{\varphi_{0}}{2}}+y_{2}\times \sin\alpha \right)\times \frac{B}{{\left(y_{1}-y_{2}\right)}^{2}}=\\ & =\pm \frac{D}{y_{1}-y_{2}} \end{array}$$

The quantization uncertainty ΔD is a discrete function of $\left(y_{1}-y_{2}\right)\in N^{+}$ and $y_{2}\in N^{+}$. Since ΔD depends also on the value of $y_{2}$, it means that the uncertainty increases not only with distance *D*, but also with object altitude *H*. The quantization uncertainty of *H* depends on the distance estimation and may be considered per analogiam.

**Figure 4:**
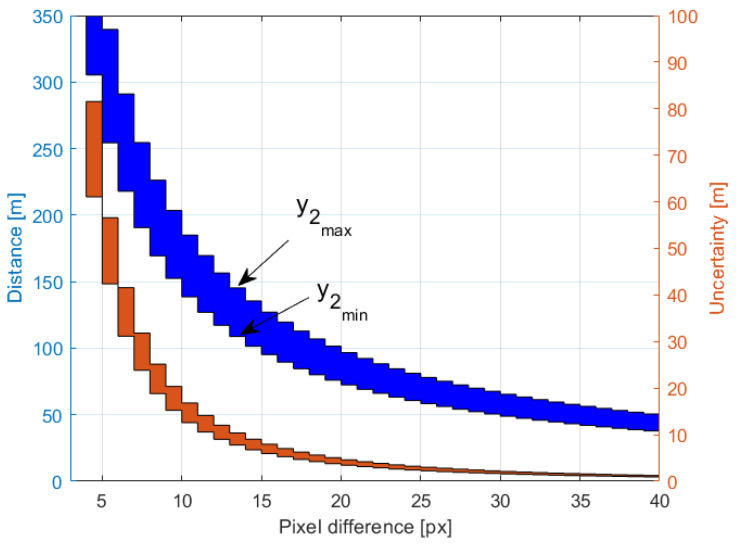
The distance measurement and its uncertainty for varying $\left(y_{1}-y_{2}\right)$ where $y_{2_{max}}$ and $y_{2_{min}}$ depict the numbers of the top and bottom pixel, respectively, on the $C_{2}$ image plane.

Figure 4 shows how for a varying pixel difference, $\left(y_{1}-y_{2}\right)$, the quantized value of distance measurement *D* and its uncertainty ΔD depend on the $y_{2}$ value, which is a measure of object elevation. The simulations for the highest $y_{2_{max}}$ and lowest altitude $y_{2_{min}}$ were performed for $y_{0}$ = 1440 px and $\varphi_{0}$ = $48.8^{\circ }$, corresponding to the off-the-shelf IMX219 camera with a focal lens of f=3 mm and a large baseline *B* = 1 m [10].

**Figure 5:**
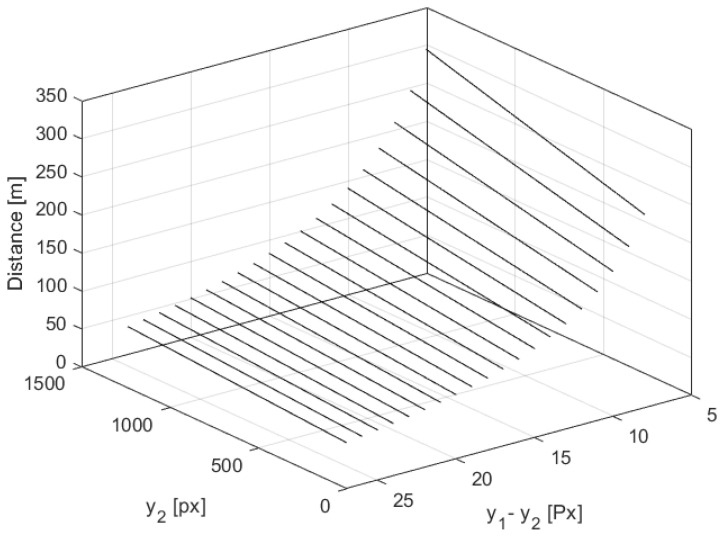
The distance measurement as a function of the pixel difference, $\left(y_{1}-y_{2}\right)$, and object position on the $C_{2}$ image plane, $y_{2}$, within a range of 300 m.

**Figure 6:**
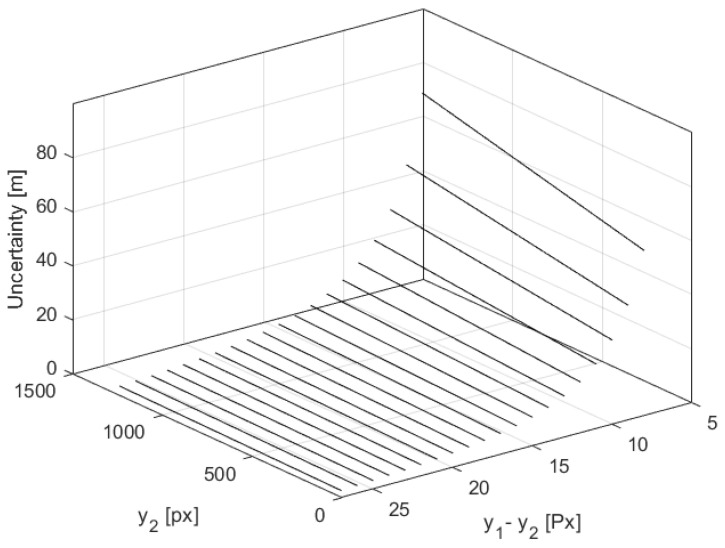
The quantization uncertainty of the distance measurement as a function of the pixel difference, $\left(y_{1}-y_{2}\right)$, and object position on the $C_{2}$ image plane, $y_{2}$, within the range of 300 m

Figure 5 and Figure 6 illustrate how the quantized distance measurement *D* and its quantization uncertainty ΔD respectively depend on the pixel difference, $\left(y_{1}-y_{2}\right)$, and object position on the $C_{2}$ image plane, $y_{2}$. The simulation was done within the range of 300 m. It proves that in the worst case, quantization uncertainty ΔD could be of 70 m, which gives a measurement uncertainty of 35 m.

### 5.2. System Processing

**Figure 7:**
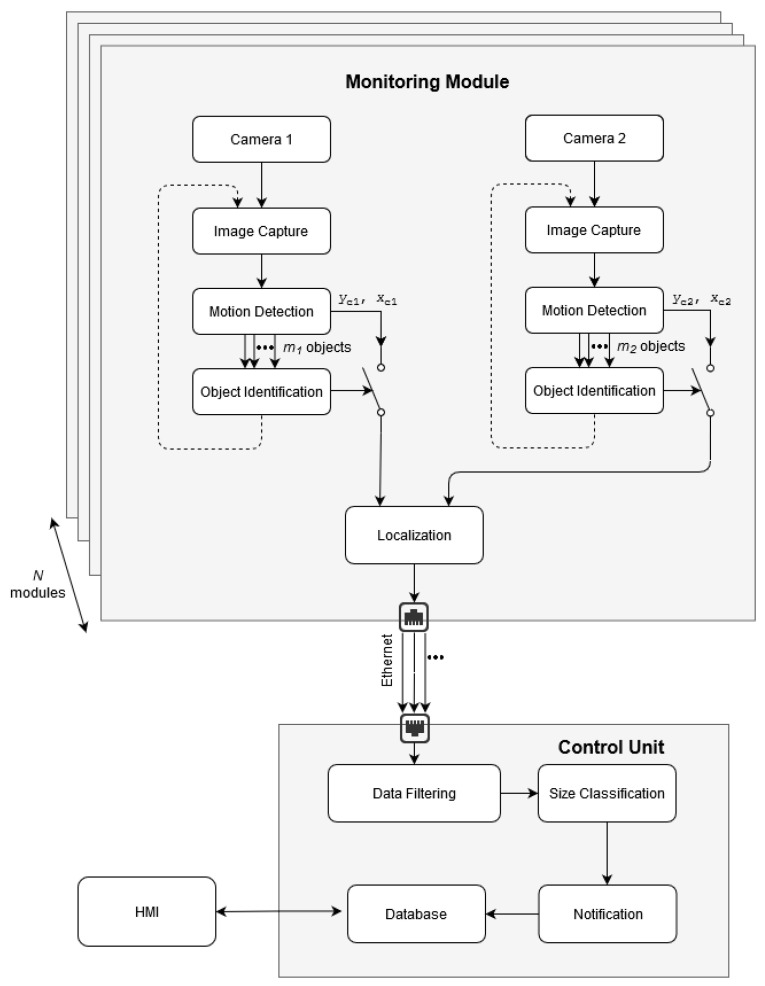
General system processing architecture consisting of *N* monitoring modules and the control unit.

Figure 7 presents the processing architecture of the system, which is based on the distributed computing and IoT paradigms. The proposed architecture supported by a stable Ethernet connection enables reliable real-time communication between the *monitoring modules* where images are collected and the *control unit* where the measurement data are processed.

The *monitoring modules* with the on-board LPU provide the video streaming from the stereovision set consisting of two cameras. The flying bird identification is based on the motion detection and object identification algorithms presented in the authors’ previous studies [10]. The CNN distinguishes bird-like objects from sky artifacts such as clouds, snow, rain, etc. When a detected moving object is identified as a bird, a warning trigger is activated, and the information from the *motion detection algorithm* including the estimated object’s center coordinates, $x_{c}$ and $y_{c}$, is sent to the 3D *localization unit*. The optimization procedure of the detection and identification algorithm was described in the authors’ previous work [10].

Via Ethernet, the control unit receives information including the object’s 3D positioning along with the image miniature and object contour [10]. In the *data filtering* block, a statistical analysis is performed to conclusively distinguish birds from other bird-like objects such as the drones, airplanes, and insects. Then, based on data about the object width, height, and contour received from the *motion detection* algorithm, as well as the estimated distance calculated in the *localization algorithm*, the *size classification algorithm* estimates the object’s size to sort it into one of three categories: *small, medium,* or *large* [10]. After classification, the notification protocol via HMI is provided to the users’ apps and archived in a local database. The deterrence module could be activated if needed.

### 5.3. Size Classification

Knowing the distance *D* and the size of a detected object on the image plane as $p_{W}$ (px) and $p_{H}$ (px) [10], the bird’s wingspan $P_{W}$ (m) and height $P_{H}$ (m) could be estimated from:

(12) $$\left\{\begin{array}{c} P_{W}=D\times p_{W}\times \frac{SIA}{f}\times \frac{1}{y_{0}}\\ P_{H}=D\times p_{H}\times \frac{SIA}{f}\times \frac{1}{y_{0}} \end{array}\right.$$

where *SIA* is the camera’s Sensor Image Area.

**Figure 8:**
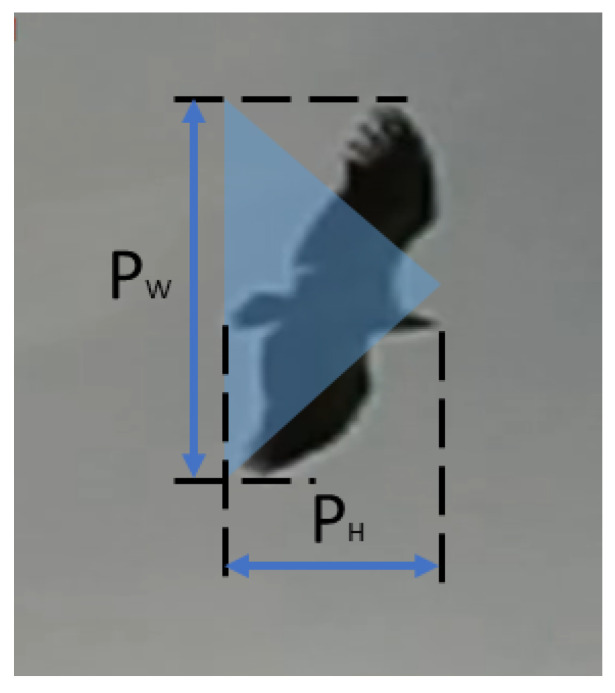
Graphical illustration of the bird’s size estimation.

Previous studies showed that the approximation of the bird’s size with an isosceles triangle enables classification of its size as small, medium, or large [10]. Figure 8 illustrates how the bird size could be estimated. The triangle base corresponds to the bird’s wingspan $p_{W}$ (px), and the height of the triangle $p_{H}$ (px) denotes the bird’s height. Then, the triangle area $O_{approx}$ is a measure of the bird’s size.

**Figure 9:**
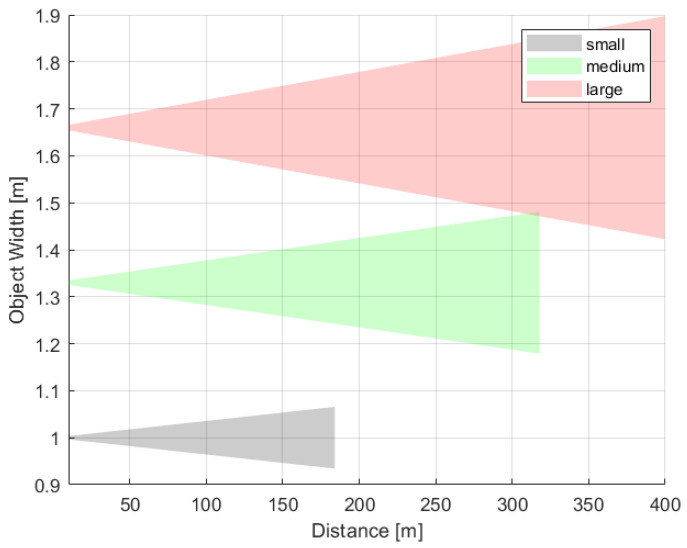
The quantization uncertainty of $P_{W}$ with respect to the distance. The calculations were made for average birds representative of each class, i.e., 1 m, 1.32 m, and 1.67 m wingspans representative of small, medium, and large bird classes, respectively.

Since the representation of an object on an image depends significantly on the object distance from the *monitoring modules*, then the size classification accuracy depends on the quantization error. The uncertainties of the measurement of $P_{W}$ as a function of the distance for typical small, medium, and large objects are presented in Figure 9. Within the requested distance ranges, there are no overlaps between the shown classes; however, the fuzziness resulting from the distance measurement uncertainty could be observed for birds of sizes close to the inter-category boundaries. The presented simulations were performed for the parameters selected in Section 5.1, and the SIA was set to 3.76 mm, which corresponds to the Sony IMX219 sensor. The calculations were done for average birds representative of each class, i.e., 1 m, 1.32 m, and 1.67 m wingspans for small, medium, and large, respectively. The measures of $P_{H}$ and $O_{approx}$ show similar uncertainty and may be considered per analogiam.

The estimate of object area $O_{approx}$ is used for the classification of the birds [10] into three categories, with the boundary values, $O_{b1}$ and $O_{b2}$, which were defined based on ornithologists suggestions. The common buzzard and the red kite were selected as boundary representatives of the medium and large objects. Therefore, each object smaller than $O_{b1}=0.22$ m${}^{2}$, corresponding to the size of the common buzzard, is considered as small, and each object bigger than or equal to $O_{b2}=0.48$ m${}^{2}$, corresponding to the size of the red kite, is considered as a large object. The calculation of boundary values of $O_{b1}=0.22$ m${}^{2}$ were performed for wingspans of 1.1 m and 1.45 m and heights of 0.4 m and 0.66 m, for the common buzzard and red kite [69], respectively.

## 6. Prototyping

This section firstly considers the optimization of the parameters within a range of constraints stated in Section 4, and then, the prototype of the system is presented.

### 6.1. Parameter Optimization

**Figure 10:**
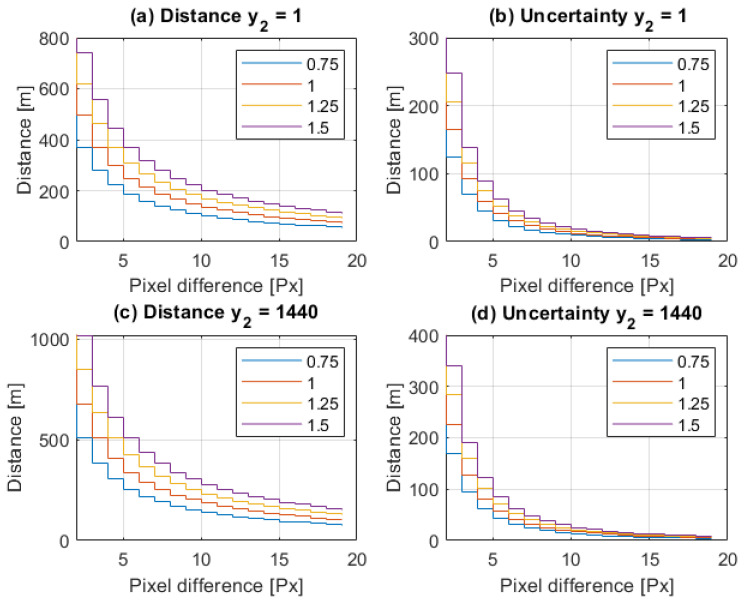
The distance measurement and its uncertainty as a function of the pixel difference for baseline *B* = (0.75, 1, 1.25, 1.5) m for different object placements on the image plane: $y_{2}$ = 1 in (**a**) and (**b**); $y_{2}$ = 1440 in (**c**) and (**d**).

From (8) and Figure 10, it can be seen that the core structural parameters of the proposed method are: the baseline, *B*, image resolution, $y_{0}$, and FoV, $\varphi_{0}$; therefore, the selection of their values is crucial.

A camera image resolution $y_{0}=1440$ px was selected due to the limitation of the computational complexity of the applied algorithms and the corresponding capabilities of the local processing units. Camera’s focal length *f* and its FoV defined by $\varphi_{0}$ are interdependent. Previous studies [10] showed that the maximum possible FoV can be realized using the IMX219 with a focal lens of f=3 mm and an FoV $\varphi_{0}=48.8^{\circ }$.

As a rule of thumb, the spatial vision is correct when the baseline is between 1/100 and 1/30 of the system range [70]. However, due to technical reasons, the baseline should not exceed 1.5 m. To select an acceptable baseline length, an evaluation of the distance measurement and its uncertainty was dione. The simulation results of *D* and ΔD for the object image detected at the top and at the bottom of the camera matrix were collected for *B* = {0.75 m, 1 m, 1.25 m, 1.5 m}; see Figure 10. From their analysis, it can be concluded that in the worst case at 300 m, ($y_{1}-y_{2}$) = 4 px and ($y_{2}$) = 1440 px, with measurement quantization uncertainty ΔD = ±81 m for *B* = 1 m, and for *B* = 1.5 m, ΔD = ±61 m. Therefore, the stereoscopic baseline *B* = 1 m was selected as fulfilling the requirement for a 10% localization accuracy with the shortest baseline *B*.

### 6.2. Hardware Prototyping of the Monitoring Modules

**Figure 11:**
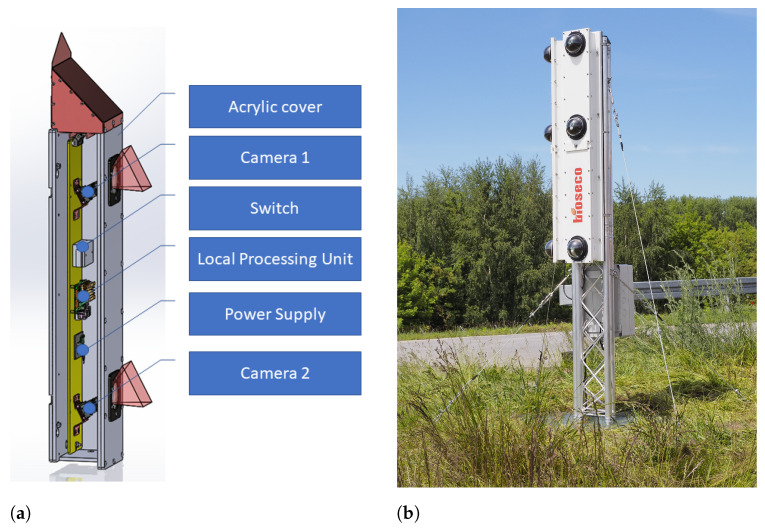
(**a**) The prototype computer drawing of the monitoring module; (**b**) the system installation composed of three monitoring modules and one control unit.

**Figure 12:**
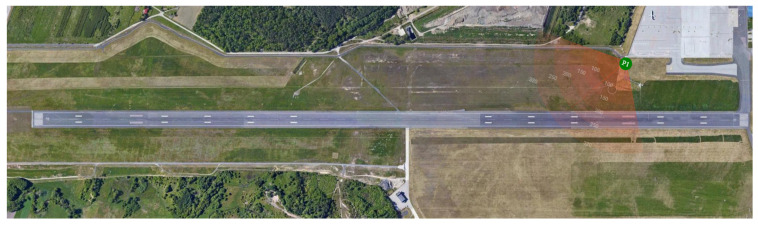
The installation spot depicted as P1 at the airport.

The prototype of the *monitoring modules* is presented in Figure 11, and the installation spot is shown in Figure 12. Each module was composed of two IMX219 cameras with a f=3 mm lens, having a vertical FoV $\varphi_{0}=48.8^{\circ }$ and allowing the image capture with a resolution of $y_{0}=1440$ px. To optimize the monitoring space, the rotation angle of the system (optical axes of both detection cameras) was set to $\alpha =\varphi_{0}/2$. The computational core of the LPU was an ARM v8.2 processor with 8 GB RAM and 384 CUDA cores and 48 Tensor cores for the AI based object *identification algorithm*. The *monitoring modules* were equipped with a switch allowing the IoT configuration. To ensure low weight, the system was composed of an acrylic cover.

**Figure 13:**
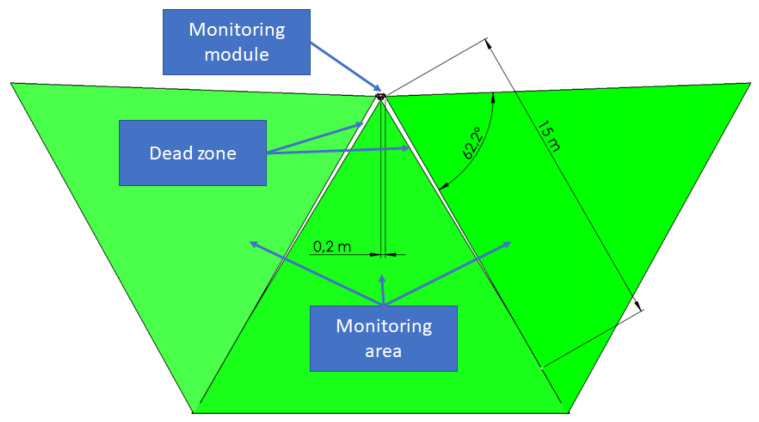
The monitoring area and dead zone configuration of the system prototype consisting of three monitoring modules.

The prototype of the system included an auxiliary recording camera allowing real-time video streaming and recording for verification and validation of the detection system. The configuration of three *monitoring modules* allowed monitoring of the area within the field of view of $\varphi =180^{\circ }$, as shown in Figure 13, where small dead zones near construction could be neglected as having no impact on the detection efficiency.

The *control system* ran on a database Dell server equipped with 3.6 GHz Xeon X5687 processor and 8 GB of RAM. As the memory storage, two 8 TB hard drives were used. The connection between the *monitoring modules* and the *control system* was provided by the Ethernet protocol. The *monitoring modules* were powered by safety extra-low voltage.

## 7. Validation and Testing

**Figure 14:**
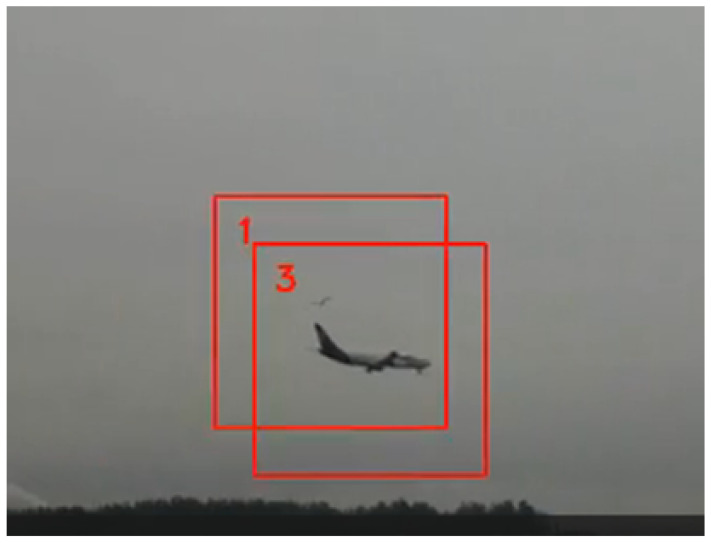
A picture from the monitoring module during the tests—a bird with an airplane in a risk situation. Frame 1 and Frame 3 are centered on the detected bird and the airplane, respectively.

The system prototype was installed on a dedicated stand in a test field, which was a flat open space near the runway of Reymont Airport in Lodz, Poland (IATA: LCJ, ICAO: EPLL), as shown in Figure 11 and Figure 12. The prototype was equipped with three *monitoring modules* and one *control unit*. Mutual placement of the stereoscopic cameras was manually set based on the fixed distant object. The positions of the images were manually determined using transformation by handle in the GIMP software. The system reported approaches by birds in flight, and an example of one such observed dangerous approach of a bird with an airplane is presented in Figure 14.

**Figure 15:**
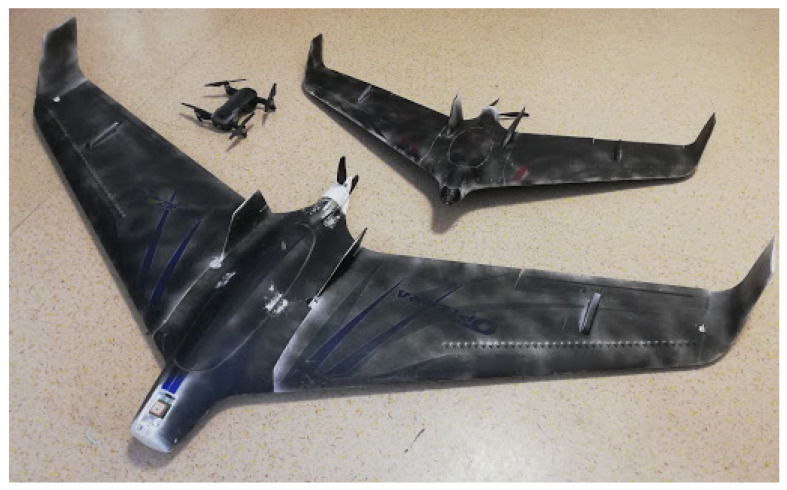
Small, medium, and large drones used during the validation of the system.

For the quantitative evaluation of the system performance in terms of detection efficiency and localization precision, bird-like drones equipped with GPS recorders were used. Two fixed-wing drones and one quadrocopter representing small, medium, and large objects are presented in Figure 15, and their dimensions provided by the manufacturer in terms of the wingspan, height, and total area are shown Table 3. The drones were programmed to fly along a given path within the system vicinity.

To evaluate the system detection efficiency, test flights for the three drones were performed. The drones flew at a random speed and altitude within the desired system detection range. The system detected the small drone 1565 times, the medium drone 2248 times, and the large drone 2875 times, during the 3 min, 12 min, and 10 min flights, respectively. The detection efficiency presented in Table 4 was calculated as the relationship between the time when the drone was visible to the monitoring module and the time of flight in the defined range. Table 4 summarizes the results. The presented results prove that the desired efficiency was achieved within the requested detection range defined in Table 1.

To quantitatively evaluate the developed system’s ability to carry out 3D object localization, it was tested in nine different scenarios defined in Table 5. The drones were turned on in autopilot mode using the remote controller, and they flew around the module at a predefined approximately constant distance and altitude, with different distances *D* and altitudes *H* used for different scenarios. The subscripts *S*, *M*, and *L* included in the scenarios listed with Roman numerals denote small, medium, and large drones, respectively. The average speed of the small, medium, and large drones during each test was 4.0 m/s, 20.0 m/s, and 15.0 m/s, respectively.

For each test flight, the mean distance $\bar{D}$, height $\bar{H}$, and corresponding standard deviation $\sigma_{\bar{D}}$ and $\sigma_{\bar{H}}$, for GPS and detection module data, respectively, were estimated. The GPS measurements were treated as reference values for the analysis of system uncertainty presented in the last four columns, where $\Delta \bar{D_{k}}$ and $\Delta \bar{H}$ depict the mean absolute accuracy of the distance and height measure, respectively, and $\delta \bar{D_{k}}$ and $\Delta \bar{H}$ depict the corresponding relative accuracy of the distance and height measure, respectively.

**Figure 16:**
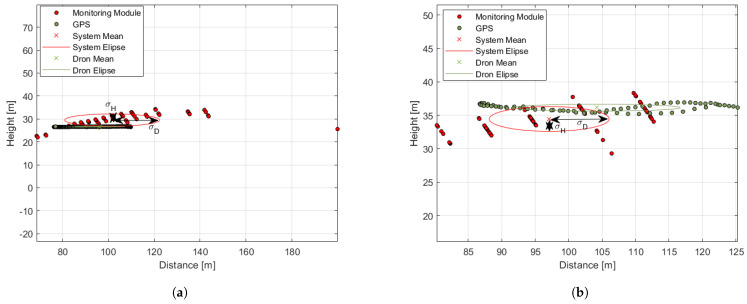
The graphical illustration of the tests results. Distance from a *monitoring module* vs. the height of the drone during the test. Green dots: GPS data; red dots: data from the module. The corresponding color ellipses illustrate the standard deviations of the respective distance and height measurements. (**a**) Scenario III; (**b**) Scenario IV.

**Figure 17:**
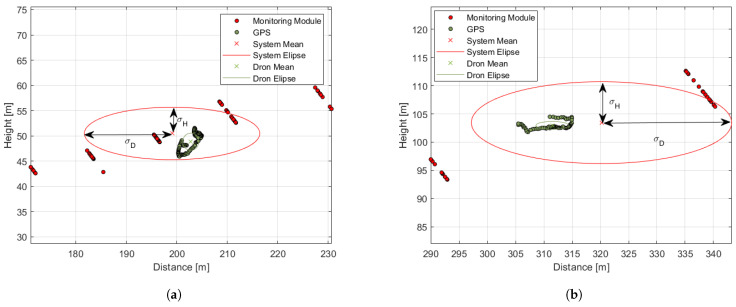
Graphical representation of the tests results. Distance from a *monitoring module* vs. the height of the drone during the test. Green dots: GPS data; red dots: data from the module. The corresponding color ellipses illustrate the standard deviations of the respective distance and height measurements. (**a**) Scenario VI; (**b**) Scenario IX.

Examples of the graphical illustration of the test results are presented in Figure 16 and Figure 17 for the small, medium, and large drones, respectively. The flight scenarios were chosen to show the system capabilities at the detection range borders for each drone. The green and red dots represent localization measurement samples from the GPS and from the system, respectively. The ellipses illustrate the measurement statistics where their center coordinates, $X(\bar{D},\bar{H})$, correspond to the mean values of the distance and height measurements, respectively. Their semi-major axes depict the standard deviation $\sigma_{\bar{D}}$, and the semi-minor axes correspond to the standard deviation $\sigma_{\bar{H}}$.

At long distances of more than 200 m, the quantization error of a single measurement was greater than the desired localization precision. However, statistics allow the reduction of the quantization error, which meets the user’s desire; see Table 1. The mean values of the distance and height uncertainty dropped below the expected 10% even for far distances of more than 300 m, which is above the quantization uncertainty of the distance measurement; see Figure 6. The system detection range and localization precision depend on the object size. The system was able to detect the small drone from 100 m, the medium drone up to 200 m, and the large drone up to 300 m.

Table 3 includes the test drones’ data sheet information, which were treated as reference values. Table 6 shows the test results for the size estimates and their quality along with the results of bird classifications, and they are presented in the last three columns of the table. For each scenario defined in Table 5, the drones’ width, $\bar{P_{w}}$, height, $\bar{P_{h}}$, and size, ${\bar{O}}_{approx}$, were estimated from the images, and then, the estimates’ variances $\sigma_{\bar{P_{w}}}$, $\sigma_{\bar{P_{h}}}$, and $\sigma_{{\bar{O}}_{approx}}$ were calculated, respectively. Despite relatively high estimation uncertainties, the system was capable of classifying drones into their correct categories.

Object classification into one of three categories of small, medium, and large was based on the estimate $O_{approx}$ and defined heuristically. The selected boundaries between categories were: between small and medium $O_{b1}=0.22$ m${}^{2}$ and between medium and large $O_{b2}=0.48$ m${}^{2}$, as introduced and presented in Section 5.3. The test results proved that within the desired ranges, the system classified small and large objects with a reliability of 99.6% and 91.4%, respectively. The classification reliability of medium objects was 65.4%. Nevertheless, medium objects were more likely to be classified as large (25.4%) rather than small (9.0%), which errs on the safe side from an application point of view. It is worth noting that the classification of objects should be treated as a fuzzy categorization, because the real sizes of birds of the same species vary. Furthermore, size estimates are biased by measurement uncertainties. Nevertheless, the test results confirmed that the average size ${\bar{O}}_{approx}$ calculated for each scenario allowed the evaluation of the object size correctly in each case.

## 8. Discussion, Conclusions, and Future Work

This work proposes a stereovision based detection system for monitoring the space near airports to identify and localize moving objects. The system is a reliable and cost-effective solution for the prevention of bird strikes around airport runways.

A new stereovision structure is proposed, composed of two cameras coupled in stereovision mode, with the cameras’ optical axes able to be freely oriented to cover the desired monitoring space from one installation spot within the cameras’ common FoV. A set of detection modules could extend the system observation FoV up to 360${}^{\circ }$. One can estimate that a medium size airport with a 2600 m runway can be covered using up to seven systems, each equipped with eight monitoring modules. The system software configuration based on the distributed computing concept powered by machine learning algorithms embedded in the IoT paradigm ensures real-time performance. Apart from the detection of moving objects, the system is capable of localizing and classifying them based on their size.

To make the system desirable and flexible for different airport sizes, the user-driven design was applied, which included many actors such as airport stakeholders, local and ecological authorities, designers, and future users. This has driven the design solution into a customizable system, which ensures cost-effectiveness without compromising system reliability.

The system was modeled and optimized using MATLAB software. The evaluation method included the analysis of the localization uncertainty and enabled system optimization. The quantitative evaluation of the system performance showed that the proposed solution meets the desired requirements regarding detection range and localization precision.

The modeled system was implemented and prototyped and then installed in a test field, which was a flat open space near the runway of Reymont Airport in Lodz, Poland. To validate the system performance, two drone sizes of 2.0 m and 1.2 m and one quadrocopter of 0.24 m were applied, imitating large, medium, and small birds, respectively. Nine test scenarios, three for each device, were applied to prove system localization and size estimate accuracy, as well as to prove the detection efficiency and ability to correctly classify the objects.

The tests proved that the system detects small objects within a range of 100 m with an efficiency of 94%. Medium objects can be detected within a range of 250 m with an efficiency of 93%, whereas the large object detection range of 300 m had a detection efficiency of 93%; see Table 4.

The estimates of the localization uncertainty for both distance and height measurements varied from 0.7% up to 9.7%, but did not exceed the required 10%, as shown in Table 5.

Estimations of drone size, which is used for object classification, were done for all nine scenarios; see Table 6. The test results proved that the system is capable of distinguishing small and large objects with a reliability of 99.6% and 91.4%, respectively. The classification reliability of medium objects was 65.4%. The results show that the approximated sizes were overestimated compared to the reference ones. However, this type of result is not fatal, and the applied classification algorithm is able to sort the objects into the correct categories. Nevertheless, the test results confirmed that by means of statistics, it is possible to enhance the object’s size estimation.

The system validation proved that the system implements all the desired functionalities and fulfills all the regulatory requirements and therefore can be used for standalone autonomous bird monitoring, complementing ornithologists’ work to minimize the risk of bird collisions with airplanes.

Among other future developments, a tracking algorithm to anticipate bird flight paths could be implemented to improve system reliability and localization accuracy. The implementation of Multiple Hypothesis Tracking (MHT), Kalman filter, or Probability Hypothesis Density (PHD) are considered as possible solutions. Moreover, the classification could be extended to include the recognition of bird species, which could improve long-term wildlife monitoring. Other possible work may also concern the detection of mammals or Foreign Object Debris (FOD) within an airport’s proximity.

Furthermore, ornithological long-term observations should be performed to verify the system performance in terms of bird detection efficiency and false positive rate. These observations could also validate the system performance in overcast weather conditions, which would be required before its implementation at airports in autonomous operational mode.

The precise calibration of a large-base stereovision system is complex and may cause a large positioning uncertainty [74]. Therefore, our future work will focus on an autonomous in situ calibration of the system.

Aviation safety at airports requires also the detection of FOD, as well as land mammals. The monitoring area of the proposed detection system could be extended to cover the whole runway.

Future work may also concern the deployment of a multi-module configuration along an airport’s runway to ensure full coverage of the skies within an airport’s legal jurisdiction.

## Figures and Tables

**Table 1:** UDD summary: functionalities, constraints, and applied technologies and algorithms.

Functionalities		Particular Constraints	Technologies and Algorithms Used
General	Itemized		
Real-time runway monitoring	Detection and identification of moving objects	Reliability ≥98%, latency ≤5 s, computation rate > 15 FPS, FP rate < 5%, robustness for weather and light conditions	Stereovision, motion detection, machine learning (convolutional NN), distributed computing, microcontroller
	Localization, positioning	3D positioning ranges: large (red kite) (20 m–300 m), medium (common buzzard) (20 m–200 m), small (swallow) (20 m–75 m), corresponding wing image size (10 px–10,000 px), localization uncertainty < 10%	
Classification/management	Object classification	Bird/no-bird, small/medium/large bird, reliability > 80%, simultaneously up to 4 individual, birds, and/or flocks	Multi-dimensional signal processing, distributed computing, machine learning (convolutional and deep NN), strobe, audio
	Threat classification	According to the airport’s specific horizontal and vertical zones	
	Bird risk management	Manual and automated repelling, cannot distract people, especially pilots	
HMI	System accessibility	Redundant reliable link with error warning 24/7, using web/mobile app to view current and archive events, manual activating repelling system	Linux/MacOS/Windows/Android/iOS, Edge/Chrome/Mozilla/Safari, MySQL, ReactJS, Ethernet, Wi-Fi, TCP/IP
	Event traceability, archiving	Automate and periodical reporting (monthly, quarterly, annually), compliant with the ICAO and the EASA regulations, manual reporting of eyewitness observations	
Affordability	Customizability, versatility	Customize monitoring range and observation zones, customize object classification	Distributed computing, IoT, CUDA, GPU, modularity
	Cost-effectiveness	System price, easy installation, and replacement		
	Serviceability, /operability	Automated and on-demand online, in situ auto-test, in situ auto-calibration	

**Table 2:** Definition of the basic variables and parameters of the mathematical model along with units used.

Symbol	Name	Unit
*B*	Baseline, the line segment connecting the cameras’ centers.	(m)
$D_{b}$	The length of the segment connecting the object with the line through the centers of the two cameras along a line parallel to the optical axes of the cameras.	(m)
$D_{k}$	The distance of a detected object to a line through the centers of the two cameras.	(m)
$b_{0}$	The intersection point of the line of $D_{b}$ with the baseline.	(-)
$b_{1}$	The distance from the first camera C1 to $b_{0}$ in a direction perpendicular to the optical axes of the cameras.	(m)
$b_{2}$	Th distance from the second camera C2 to $b_{0}$ in a direction perpendicular to the optical axes of the cameras.	(m)
*D*	The distance from the first camera to the plane of the object, wherein the plane of the object is a plane perpendicular to the optical axes of the cameras.	(m)
$d_{1}$	The distance from the first camera C1 to $b_{0}$ in a direction parallel to the optical axes of the cameras.	(m)
$d_{2}$	The distance from the second camera C2 to $b_{0}$ in a direction parallel to the optical axes of the cameras.	(m)
$\varphi_{0}$	The cameras’ field of view.	(${}^{\circ }$)
$\varphi_{1}$	The angle between the projection line of the object on the first camera C1 and the optical axis of the first camera.	(${}^{\circ }$)
$\varphi_{2}$	The angle between the projection line of the object on the second camera C2 and the optical axis of the second camera.	(${}^{\circ }$)
α	The rotation angle defined as an angle between the (parallel) optical axes of the cameras and the base line. The rotation of the first camera C1 is around the first axis, perpendicular to the optical axis of the first camera, and the rotation of the second camera C1 is around the second axis, parallel to the first axis.	(${}^{\circ }$)
$y_{0}$	The cameras’ resolution along the Y axes wherein the Y axis of a camera is perpendicular to the rotational axis of the camera (the first axis for the first camera and the second axis for the second camera) and within the image plane of the corresponding camera.	(px)
$y_{1}$	The pixel number of the object’s center projection on the image plane of the camera C1 along the Y1 axis wherein the Y1 axis is perpendicular to the rotational axis of C1 and within the image plane of the camera.	(px)
$y_{2}$	The pixel number of the object’s center projection on the image plane of the camera C2 along the Y2 axis wherein the Y2 axis is perpendicular to the rotational axis of C2 and within the image plane of the camera.	(px)

**Table 3:** Parameters of drones used for the tests.

Parameter	Small [71]	Medium [72]	Large [73]
Wingspan	0.24 m	1.20 m	1.99 m
Height	0.10 m	0.53 m	1.04 m
Total area	0.012 m${}^{2}$	0.28 m${}^{2}$	0.67 m${}^{2}$
$O_{aprox_{ref}}$	0.04 m${}^{2}$	0.32 m${}^{2}$	1.02 m${}^{2}$
Requested detection range	75 m	200 m	300 m

**Table 4:** Evaluation of system detection efficiency.

	Small			Medium			Large		
Detection Range	Detection Time	Flight Time	Detection Efficiency	Detection Time	Flight Time	Detection Efficiency	Detection Time	Flight Time	Detection Efficiency
	(s)	(s)	(%)	(s)	(s)	(%)	(s)	(s)	(%)
(0–50>	29	32	91	26	26	100	-	-	-
(50–100>	80	85	94	82	82	100	-	-	
(100–150>	20	40	50	226	235	96	24	24	100
(150–200 >	-	-	-	322	329	98	242	252	96
(200–250>	-	-	-	64	69	93	102	105	98
(250–300>	-	-	-	-	-	-	26	28	92
(300–350>	-	-	-	-	-	-	206	362	57

**Table 5:** Test plan of the designed system. *N* is the number of samples registered during the test, and the error is the difference in the mean between the GPS and the system measurements.

	GPS Data				Detection Module Data					Uncertainty			
**Scenarios**	${\bar{D}}_{kGPS}$	$\sigma_{\bar{D_{kGPS}}}$	${\bar{H}}_{GPS}$	$\sigma_{\bar{H}}$	***N***	${\bar{D}}_{k}$	$\sigma_{\bar{D_{k}}}$	$\bar{H}$	$\sigma_{\bar{H}}$	$\Delta \bar{D_{k}}$	$\Delta \bar{H}$	$\delta \bar{D_{k}}$	$\delta \bar{H}$
	**(m)**	**(m)**	**(m)**	**(m)**	**(-)**	**(m)**	**(m)**	**(m)**	**(m)**	**(m)**	**(m)**	**(%)**	**(%)**
I${}_{S}$	46.4	0.3	26.7	0.2	85	45.6	1.6	26.9	0.6	0.8	0.2	1.7	0.7
II${}_{S}$	66.6	2.2	32.0	0.2	86	68.7	3.7	34.6	1.4	2.1	2.6	3.2	8.1
III${}_{S}$	96.3	10.8	26.7	0.1	82	101.7	20.7	29.3	2.7	5.4	5.9	5.6	9.7
IV${}_{M}$	104.2	12.5	36.1	0.5	99	97.1	8.9	34.5	1.8	7.1	1.6	6.8	1.6
V${}_{M}$	133.6	13.7	38.5	2.3	157	141.3	9.6	35.4	3.8	7.7	3.1	5.0	8.0
VI${}_{M}$	202.8	1.6	48.8	1.7	97	199.1	17.4	50.5	5.1	1.7	6.7	1.8	3.4
VII${}_{L}$	129.3	2.5	53.0	0.7	137	139.4	9.5	53.7	3.6	10.1	0.7	7.8	1.3
VIII${}_{L}$	202.9	4.9	96.9	0.7	101	186.7	16.8	88.3	6.7	16.2	8.6	7.9	8.8
IX${}_{L}$	311.5	2.9	102.9	0.7	31	320.2	23.0	103.5	7.2	8.7	0.6	2.7	0.5

**Table 6:** The test results of drone size estimations, where the reference sizes can be seen in Table 3. The classification boundaries are $O_{b1}=0.22$ m${}^{2}$ and $O_{b1}=0.48$ m${}^{2}$.

	Detection Module Data						Uncertainty			Classification		
**Scenarios**	$\bar{P_{w}}$	$\sigma_{\bar{P_{w}}}$	$\bar{P_{h}}$	$\sigma_{\bar{P_{h}}}$	${\bar{O}}_{approx}$	$\sigma_{\bar{O_{approx}}}$	$\Delta \bar{P_{w}}$	$\Delta \bar{P_{h}}$	$\Delta {\bar{O}}_{approx}$	**Small**	**Medium**	**Large**
	**(m)**	**(m)**	**(m)**	**(m)**	**(m** ${}^{2}$ **)**	**(m** ${}^{2}$ **)**	**(m)**	**(m)**	**(m** ${}^{2}$ **)**			
I${}_{S}$	0.32	0.04	0.17	0.01	0.035	0.002	0.08	0.07	0.005	85	0	0
II${}_{S}$	0.35	0.01	0.25	0.01	0.044	0.001	0.11	0.14	0.004	86	0	0
III${}_{S}$	0.31	0.06	0.21	0.02	0.049	0.005	0.07	0.11	0.009	81	1	0
IV${}_{M}$	1.06	0.23	0.68	0.08	0.363	0.207	0.14	0.15	0.046	8	72	19
V${}_{M}$	0.87	0.11	0.86	0.11	0.379	0.121	0.33	0.33	0.044	21	105	31
VI${}_{M}$	1.26	0.25	0.69	0.03	0.437	0.082	0.06	0.16	0.118	3	54	40
VII${}_{L}$	1.85	0.24	0.87	0.05	0.807	0.100	0.14	0.17	0.227	5	17	115
VII${}_{L}$	2.09	0.13	0.95	0.03	0.999	0.079	0.10	0.09	0.036	0	0	101
IX${}_{L}$	1.97	0.14	1.16	0.04	1.156	0.145	0.02	0.12	0.121	0	1	30

## Data Availability

The presented data are accessible for authorized staff according to the local regulation.

## Abbreviations

The following abbreviations are used in this manuscript:

ICAO	International Civil Aviation Organization
EASA	European Union Aviation Safety Agency
WHM	Wildlife Hazard Management
IoT	Internet of Things
WBA	World Birdstrike Association
CNN	Convolutional Neural Networks
UDD	User-Driven Design
FoV	Field of View
SIA	Sensor Image Area
HMI	Human Machine Interface
LPU	Local Processing Unit
